# Triptolide induces apoptosis of breast cancer cells via a mechanism associated with the Wnt/β-catenin signaling pathway

**DOI:** 10.3892/etm.2014.1729

**Published:** 2014-05-26

**Authors:** HONGMIN SHAO, JINGHUA MA, TIANHUA GUO, RONGRONG HU

**Affiliations:** Department of Oncology, Hospital of Traditional Chinese Medicine, Yantai, Shandong 264000, P.R. China

**Keywords:** triptolide, breast cancer, Wnt, β-catenin

## Abstract

Triptolide is a diterpene triepoxide compound extracted from the medicinal plant, *Tripterygium wilfordii* Hook F. The aim of the present study was to determine whether triptolide inhibits the proliferation of breast cancer cells and to further investigate the associated molecular mechanisms. The effects of triptolide on the cell viability of three breast cancer cell lines, specifically, highly metastatic MDA-MB-231, human epidermal growth factor receptor 2-positive BT-474 and estrogen receptor-positive MCF7 cells, were measured using 3-(4,5-dimethylthiazol-2-yl)-2,5-diphenyltetrazolium bromide and apoptosis assays. Western blot analysis was performed to investigate the expression levels of β-catenin in the control and triptolide-treated cells. The results demonstrated that triptolide treatment caused cell death in the three types of malignant cell lines. Treatment with 25 nM triptolide for 48 h exhibited marked inhibitory effects on the cell viability of the three types of cells, with greater effects observed in BT-474 cells compared with the other two cell types. When compared with the cells not treated with triptolide, 50 nM triptolide treatment resulted in apoptosis of MDA-MB-231, BT-474 and MCF7 cells with apoptotic rates of ~80%. Western blot analysis indicated that triptolide treatment of MDA-MB-231, BT-474 and MCF7 cells decreased the expression levels of β-catenin to 5–10% of the levels observed in the cells treated with dimethyl sulfoxide only. Therefore, the results of the present study indicate that triptolide induces the apoptosis of breast cancer cells via a mechanism associated with the Wnt/β-catenin signaling pathway.

## Introduction

Triptolide is a diterpene triepoxide antibiotic compound that can be isolated from extracts of the medicinal plant, *Tripterygium wilfordii* Hook F, which has been used for a number of years in traditional Chinese medicine (1,2). *Tripterygium wilfordii* Hook F and triptolide have immunosuppressive and anti-inflammatory properties (3,4). Triptolide causes apoptosis by inducing the activation of caspases (5–8). Proliferation of rheumatoid synovial fibroblasts associated with rheumatoid arthritis has also been shown to decrease with triptolide treatment through a mechanism of increasing caspase-3 activity (9).

The Wnt/β-catenin signaling pathway plays important roles during the development of malignancies by regulating proliferation, migration, tissue polarity and organogenesis (10,11). In the canonical Wnt/β-catenin pathway, β-catenin functions as the central component (12). Receptor binding of Wnt inhibits the formation of a protein complex that includes axin, glycogen synthase kinase-3 and adenomatous polyposis coli (13). This inhibition leads to the accumulation of β-catenin in the cytoplasm, which then translocates to the nucleus (14). In the nucleus, β-catenin binds to T-cell factor, resulting in the transcription of target genes (15). Such aberrant β-catenin signaling has been identified in a number of types of human cancers, including melanomas, and colorectal and prostate cancers (16). β-catenin is hypothesized to affect the metastatic potential of malignant cells by changing chromatin remodeling (17) and altering the oxidative stress response (18). Therefore, reducing the constitutive activation of the Wnt/β-catenin signaling pathway is an attractive target for the treatment of cancers.

In the present study, the effects of triptolide treatment were determined on multiple breast cancer cell lines, specifically, the highly metastatic MDA-MB-231, human epidermal growth factor receptor (HER2)-positive BT-474 and estrogen receptor (ER)-positive MCF7 cell lines. Whether the anti-tumor effects of triptolide on breast cells are associated with the inhibition of β-catenin expression was also investigated. The potential of using triptolide as an effective approach for the treatment of breast cancers in the future was thus evaluated

## Materials and methods

### Reagents and cell lines

Triptolide (molecular formula, C_20_H_24_O_6_; molecular weight, 360.4 g/mol) was purchased from Santa Cruz Biotechnology, Inc. (sc-200122; Santa Cruz, CA, USA). Triptolide was dissolved in dimethyl sulfoxide (DMSO). Three breast cancer cell lines, specifically, the highly metastatic MDA-MB-231, HER2-positive BT-474 and ER-positive MCF7 cell lines, were provided by the Department of Oncology at the Hospital of Traditional Chinese Medicine (Yantai, China). MDA-MB-231, BT-474 and MCF-7 cells were cultured in Dulbecco’s modified Eagle’s medium, RPMI and α-minimal essential medium (Sigma-Aldrich, St. Loius, MO, USA), respectively. Cells were cultured at 37°C with 5% CO_2_ and 100% humidity. The medium was supplemented with 10% fetal bovine serum (FBS; HyClone Laboratories, Inc., Logan, UT, USA), 100 U/ml penicillin and 100 μg/ml streptomycin.

### 3-(4,5-Dimethylthiazol-2-yl)-2,5-diphenyltetrazolium bromide (MTT) assay

Cells at a density of 1×10^5^ cells/well in medium were placed into 6-well plates and were continuously cultured for 24 h. The cells were then treated with triptolide (0, 10, 25 and 50 nM). Cells treated with DMSO only (0 nM triptolide) were used as a control. After 48 h, cells were incubated with 0.5 mg/ml MTT (Sigma-Aldrich) for 4 h according to the manufacturer’s instructions. The cell viability of the treated cells was expressed relative to that of the cells treated with DMSO only (relative viability).

### Apoptosis assay

Cells at a density of 1×10^5^ cells/well were cultured in six-well plates in medium supplemented with 10% FBS for 24 h. This was followed by the addition of triptolide (0, 10, 25 or 50 nM). After 48 h, cells were harvested by centrifugation, rinsed twice with phosphate-buffered saline (PBS), fixed by incubation in 4% paraformaldehyde for 30 min at room temperature and then rinsed again with PBS to remove the fixative. Fixed cells were resuspended in PBS that contained 5 μg/ml Hoechst 33258 and incubated at room temperature for 15 min in the dark. The cells were placed on glass slides and examined to record the percentages of apoptotic cells with apoptotic morphology by determining the nuclear condensation and chromatin fragmentation via fluorescence microscopy (Olympus IX81, Olympus, Tokyo, Japan). To quantify the apoptotic rate, 250 nuclei from random microscopic fields were examined. Data are presented as the mean percentage of apoptotic cells relative to the total cell number.

### Western blot analysis

Cells were harvested by centrifugation and rinsed twice with PBS. Total proteins were harvested from cells, separated on 10% SDS-PAGE gels and then subjected to immunoblot analyses. Primary antibodies against β-catenin (~90 kDa) and β-actin were purchased from Santa Cruz Biotechnology, Inc. (anti-β-catenin, sc-7963; 1:200; anti-β-actin, sc-130301; 1:10,000). The secondary antibody, goat anti-mouse IgG-horseradish peroxidase, was purchased from Santa Cruz Biotechnology, Inc. (sc-2005; 1:5,000). Antibodies bound to the blots were detected using an enhanced chemiluminescence system (Pierce Biotechnology, Inc., Rockford, IL, USA). The mean optical densities (ODs) of β-catenin protein bands were normalized against the OD of the β-actin band from the same treatment. Quantity One software (Bio-Rad Laboratories, Hercules, CA, USA) was used to quantify the OD levels. Immunoblotting experiments were repeated at least 3 times.

### Statistical analysis

Experimental data are expressed as mean ± SEM. Statistical software (SPSS version 12.0; SPSS, Inc., Chicago, IL, USA) was used to perform independent sample t-tests, followed by one-way analysis of variance. P<0.05 was considered to indicate a statistically significant difference.

## Results

### Triptolide has a toxic effect on breast cancer cells

**Figure 1:**
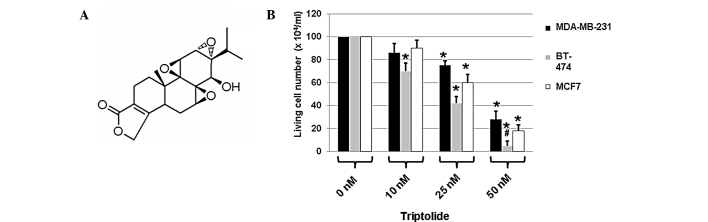
(A) Chemical structure of triptolide (molecular formula, C_20_H_24_O_6_; molecular weight, 360.4 g/mol). (B) Three breast cancer cell lines (MDA-MB-231, BT-474 and MCF7) were treated with DMSO only (0 nM triptolide) or triptolide at a concentration of 10, 25 or 50 nM. Cell viability was measured using the MTT assay after 2 days of incubation with triptolide. Values are expressed as mean ± SEM and were obtained from three independent experiments. DMSO, dimethyl sulfoxide; MTT, 3-(4,5-dimethylthiazol-2-yl)-2,5-diphenyltetrazolium bromide. ^*^P<0.05, compared with the untreated cells (0 nm); ^#^P<0.05, compared with the MDA-MB-231 and MCF7 cells that were treated with triptolide at a concentration of 50 nM.

To determine whether triptolide (Fig. 1A) exhibited toxic effects on breast cancer cells, highly metastatic MDA-MB-231, HER2-positive BT-474 and ER-positive MCF7 cells were treated with DMSO only (0 nM triptolide) or triptolide at a concentration of 10, 25 or 50 nM. Cell viability was determined using the MTT assay following treatment with triptolide. DMSO only treatment served as a control since triptolide was tested as a solution in DMSO. The cells were analyzed for differences in cell viability following the various treatments by counting the number of living cells in the presence or absence of triptolide using the MTT assay.

Results demonstrated that in comparison with the cells treated with DMSO only, 48 h-treatment with triptolide reduced the cell viability of all three types of tumor cells (Fig. 1B). Treatment with 10 nM triptolide for 48 h exhibited an inhibitory effect on the cell viability of BT-474 cells (Fig. 1B). However, treatment with 25 nM triptolide for 48 h had marked inhibitory effects on the cell viability of all three cell types, with greater effects observed in BT-474 cells compared with the other two types of cell (Fig. 1B). In addition, 50 nM triptolide treatment resulted in greater significant inhibitory effects on cell numbers (Fig. 1B). Among the three types of cell, BT-474 cells were more sensitive compared with the other two cell types (Fig. 1B). These results indicate that triptolide has significant toxic effects on breast cancer cells.

### Triptolide induces apoptosis of breast cancer cells

Since triptolide exhibited toxic effects on the highly metastatic MDA-MB-231, HER2-positive BT-474 and ER-positive MCF7 cells, the apoptotic effects of triptolide were determined in the three cell types. Cells were treated with DMSO only (0 nM triptolide) or 10, 25 or 50 nM triptolide for 48 h. To determine the apoptotic rates, a fluorescence microscopic assay was performed following the staining of the triptolide- or DMSO-treated cells with Hoechst 33258.

**Figure 2:**
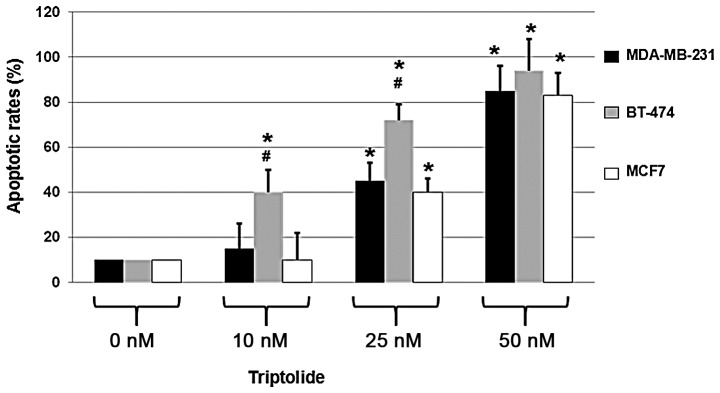
Detection of the apoptotic rates of cells treated with triptolide. Three breast cancer cell lines (MDA-MB-231, BT-474 and MCF7) were treated with DMSO only (0 nM triptolide) or 10, 25 or 50 nM triptolide. Cells were harvested at 48 h following treatment. Hoechst 33258-stained cells were examined to determine the apoptotic rates using a fluorescence microscope. Data are expressed as mean ± SEM and were obtained from three independent experiments. DMSO, dimethyl sulfoxide. ^*^P<0.05, compared with the untreated cells (0 nm); ^#^P<0.05, compared with the MDA-MB-231 and MCF7 cells that were treated with triptolide at a concentration of 10 nM or 25 nM.

As shown in Fig. 2, triptolide treatment resulted in an increase in apoptosis in all three cell types. When compared with the cells not treated with triptolide, 50 nM triptolide treatment resulted in the apoptosis of MDA-MB-231, BT-474 and MCF7 cells with an apoptotic rate of ~80%. These results indicate that the rate of apoptosis is significantly increased in triptolide-treated cells.

### Triptolide treatment results in the degradation of β-catenin

**Figure 3:**
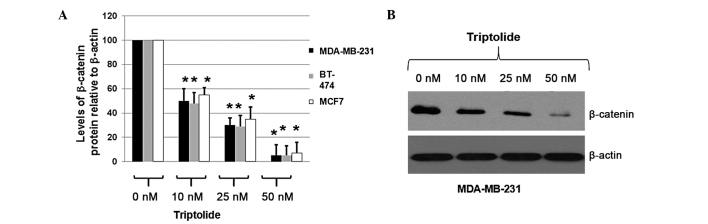
Triptolide decreases the expression levels of β-catenin. Cells were treated with DMSO only (0 nM triptolide) or 10, 25 or 50 nM triptolide and harvested after 48 h. Whole-cell extracts were prepared and immunoblot analysis was performed to analyze the expression levels of β-catenin and β-actin. Cellular β-actin was used as a loading control. (A) Levels of β-catenin proteins relative to β-actin levels in MDA-MB-231, BT-474 and MCF7 cells treated with DMSO only or 10, 25 or 50 nM triptolide. Data were obtained from at least three independent experiments. (B) A representative blot of MDA-MB-231 cell lysates. The blots of BT-474 or MCF7 cells are not shown. DMSO, dimethyl sulfoxide. ^*^P<0.05, compared with the untreated cells (0 nm).

To determine whether triptolide inhibited the expression of β-catenin in MDA-MB-231, BT-474 and MCF7 cells, the cells were treated with DMSO only (0 nM triptolide) or 10, 25 or 50 nM triptolide for 48 h. Total proteins were isolated and the expression levels of β-catenin were determined by immunoblot analysis. Cellular β-actin protein was used as a loading control. The mean OD values of β-catenin protein bands, normalized against the OD of the β-actin band from the same treatment, were calculated and subjected to statistical analyses. The calculated ratios of the levels of β-catenin proteins relative to β-actin levels were obtained and are shown in Fig. 3A.

As shown in Fig. 3A, triptolide treatment of MDA-MB-231, BT-474 and MCF7 cells decreased the expression levels of β-catenin to 5–10% of the expression levels observed in the cells treated with DMSO only, according to the calculated OD values of the β-catenin bands relative to the β-actin bands. A representative blot is shown in Fig. 3B. These results indicate that triptolide significantly decreased β-catenin expression in the breast cancer cells, suggesting that triptolide effectively inhibits the proliferation of breast cancer cells via a mechanism associated with the Wnt/β-catenin signaling pathway.

## Discussion

Triptolide is reported to have multiple functions, including immunosuppressive and anti-inflammatory properties and the induction of apoptosis via the activation of caspases (3–8). Triptolide functions by regulating multiple cellular signaling pathways, including the NF-κB pathway (19). In the present study, the inhibitory effects of triptolide were investigated in three breast cancer cell lines, specifically, highly metastatic MDA-MB-231, HER2-positive BT-474 and ER-positive MCF7 cell lines.

In the present study, triptolide treatment was found to induce apoptosis in the three types of malignant cells. Treatment with 25 nM triptolide for 48 h exhibited marked inhibitory effects on the cell viability of all three cell types, with greater effects observed in BT-474 cells compared with the other two cell types. When compared with the cells not treated with triptolide, 50 nM triptolide treatment resulted in the apoptosis of MDA-MB-231, BT-474 and MCF7 cells with apoptotic rates of ~80%. Western blot analysis indicated that triptolide treatment in MDA-MB-231, BT-474 and MCF7 cells decreased the expression levels of β-catenin to 5–10% of the expression levels observed in the cells treated with DMSO only. Therefore, the results of the present study indicate that triptolide induces apoptosis in breast cancer cells via a mechanism associated with the Wnt/β-catenin signaling pathway.

Triptolide is involved in multiple cellular signaling pathways. Recently, triptolide was shown to induce extracellular signal-regulated kinase activation and affect the generation of reactive oxygen species and the induction of endoplasmic reticulum stress via the PERK-eIF2α pathway (20). In addition, a previous study has demonstrated that triptolide induces cell cycle arrest and apoptosis in human tumor cells by inducing DNA damage and the expression of repair-associated genes (21). Therefore, the observations of the present study indicate that triptolide induces apoptosis in breast cancer cells by a mechanism associated with the Wnt/β-catenin signaling pathway, further improving the understanding of the role of triptolide as a potential treatment for breast cancer.
